# Lead-Bismuth Eutectic: Atomic and Micro-Scale Melt Evolution

**DOI:** 10.3390/ma12193158

**Published:** 2019-09-27

**Authors:** Roberto Montanari, Alessandra Varone, Luca Gregoratti, Saulius Kaciulis, Alessio Mezzi

**Affiliations:** 1 Department of Industrial Engineering, University of Rome “Tor Vergata”, 00133 Roma, Italy; alessandra.varone@uniroma2.it; 2 Elettra – Sincrotrone di Trieste, Area Science Park, 34149 Trieste, Italy; luca.gregoratti@elettra.eu; 3 Institute for the Study of Nanostructured Materials, ISMN – CNR, P.O. Box 10, 00015 Montelibretti, Italy; saulius.kaciulis@cnr.it (S.K.); alessio.mezzi@cnr.it (A.M.)

**Keywords:** liquid Pb–Bi eutectic alloy, chemical homogeneity, short range order, XPS, SPEM, HT-XRD

## Abstract

Element clustering and structural features of liquid lead-bismuth eutectic (LBE) alloy have been investigated up to 720 °C by means of high temperature X-ray diffraction (HT-XRD), X-ray Photoemission Spectroscopy (XPS) and Scanning Photoemission Microscopy (SPEM) at the Elettra synchrotron in Trieste. The short-range order in liquid metal after melting corresponds to the cuboctahedral atomic arrangement and progressively evolves towards the icosahedral one as temperature increases. Such process, that involve a negative expansion of the alloy, is mainly connected to the reduction of atom distance in Pb–Pb pairs which takes place from 350 °C to 520 °C. On an atomic scale, it is observed a change of the relative number of Bi–Bi, Pb–Pb, and Pb–Bi pairs. The Pb–Bi pairs are detected only at a temperature above ~350 °C, and its fraction progressively increases, giving rise to a more homogeneous distribution of the elements. SPEM results showed evidence that the process of chemical homogenization on an atomic scale is preceded and accompanied by homogenization on micro-scale. Clusters rich of Bi and Pb, which are observed after melting, progressively dissolve as temperature increases: Only a few residuals remain at 350 °C, and no more clusters are detected a 520 °C.

## 1. Introduction

Many physical properties of liquid metals depend on their structure, thus, its knowledge is of fundamental importance. In the past, some attempts have been made to describe the liquids as disordered crystals or dense gases, but a simple structural description of liquids, such as “periodicity” for crystals or “sparsity” for gases represents a challenge in the science of condensed matter. 

The five-fold symmetry in liquid Pb was observed through measurements of the X-rays evanescent radiation [1]. In fact, this result demonstrated that a mono-atomic liquid is made up of icosahedral blocks, as theoretically conjectured by Frank [2] and Bernal [3] more than sixty years ago. Icosahedral clusters have been then studied through different approaches and techniques, of particular relevance are molecular dynamics simulations [4,5,6] and High Temperature X–ray Diffraction (HT-XRD) [7].

The experiment of Reichert et al. [1] represents an important step towards understanding the structure of liquids; however, much remains to be done. The following issues are of particular interest for scientific knowledge and metallurgical applications: (i) The structural correlations between solid and liquid during the solidification, (ii) the phase de-mixing in metal systems with a miscibility gap in the liquid state, (iii) the liquid–liquid phase transitions in pure metals and alloys.

### (i) Structural Correlations between Solid and Liquid during the Solidification of Pure Metals

The structural correlations between solid and liquid during the solidification of some pure metals (Zn [8] and In [9,10,11]) and alloys (In-10Sn and Sn-13Pb [12,13]) have been studied in recent years by one of the present authors through HT-XRD. Other papers have been published regarding the aforesaid binary systems (see for instance [14]) and the presence of clusters in melt correlated to the crystal structure of the solid is the main established result [12,13,14,15]. 

A number of metastable phases, whose nuclei pre-exist as clusters in the liquid, has also been revealed in the Sn-Pb system by quenching from liquid [16,17,18]. Two theoretical models, based on the statistical mechanics [19] and the nano-crystalline structure [20], have been developed to explain the presence of clusters in the liquid and their changes with temperature. Lianwen Wang [21] suggested that the energy state of liquids is reasonably approximated by the energy and volume of a vacancy. 

### (ii) Phase de-Mixing in Metal Systems with a Miscibility Gap in the Liquid State

The concept of metastable micro-heterogeneity in molten alloys, which exhibit a monotectic reaction, has been pointed out in a recent review [22]. HT-XRD experiments showed that these alloys have liquid domains enriched in the two different kinds of atoms above the cupola identifying the miscibility gap. The characteristic scale of such domains was estimated to be in the range of 1–10 nm from sedimentation in a centrifuge or in natural gravity.

A large number of examples confirms the importance of thermal treatment of liquid precursors in order to improve the structure and the physical properties of numerous alloys. Higher the cooling rate during the solidification, greater the influence of melt structure on the final solid structure. Likely heat treatments of melts will become in the future an integral part of specific technological processes as heat treatments of solid alloys are today.

Important results have been obtained by investigating systems with a miscibility gap in the liquid state. Some relevant examples regard the possibility of obtaining bulk metal glasses in the systems, such as Nb–Y, where the two liquid phases can be frozen into a two-phase amorphous metallic alloy by rapid quenching [23] and the realization of Al alloys containing embedded Pb nano-dispersoids with enhanced wear behavior with respect to their coarse grained counterparts [24]. 

### (iii) Liquid–Liquid Phase Transitions in Pure Metals and Alloys

Metals in the solid-state may assume different crystalline structures depending on the temperature or pressure (polymorphic transformations). Recently, liquid–liquid phase transitions have been proved to occur in some one-component melts (e.g., Cs, Bi, Ga, Si, Ge and Se) [25,26,27,28,29,30,31] which undergo a discontinuous structure change. Discontinuous changes of the liquid structure have also been observed in some In-Sn, Pb–Bi, Pb-Sn, In-Bi binary alloys [32,33,34,35] depending on temperature change at constant pressure. The discontinuity occurs at hundreds of degrees above liquidus where there is no other defined phase line in the phase diagram. The phenomenon was revealed by HT-XRD, mechanical spectroscopy (MS) and differential scanning calorimetry (DSC). A recent paper of Zu [36] provides an overview of liquid–liquid phase transitions in metallic melts. 

Present work investigates the behavior of the liquid Pb–Bi eutectic (LBE) alloy, a good candidate as coolant and neutron spallation source in MYRRHA reactor [37]. For this application more information on atomic-scale interactions, structure and thermal physical properties, as a function of the temperature must be collected, because one of the main limitations of this reactor is the compatibility of structural materials with liquid LBE at high temperature, where corrosion and embrittlement take place.

In previous work, MS tests showed a relevant change in the microstructure of liquid LBE [38]. In the same material, a damping maximum at ~550 °C has been recorded by Zu et al. [33] and ascribed to a structural change occurring when, above a critical temperature, the alloy, that still has minor residual crystals after melting, transforms into a configuration without crystalline conglomerates. Moreover, an anomaly of the electrical conductivity σ(T) curve, observed by Plevachuk et al. [39] and Li et al. [40], was explained by considering a melt structural inhomogeneity.

This work presents the results of HT-XRD up to 720 °C, which describes the short-range order of the liquid alloy. XPS and scanning photoemission microscopy (SPEM) at the Elettra synchrotron (Trieste, Italy) with high lateral resolution [41,42] were used to investigate possible clustering of alloying elements. 

## 2. Materials and Methods 

The examined material was the Pb–Bi alloy in eutectic composition (Pb 44.1-Bi 55.9 at %). 

The alloy was prepared from high purity metals, 99.999% Pb and 99.9999% Bi (Goodfellow Cambridge Ltd, Huntingdon, UK), in an alumina crucible under an atmosphere of Ar + 5% H_2_.

HT-XRD experiments were done by using an ANTON PAAR HT–16 camera (Graz, Austria) with a sample-holder for liquid metals. The tests were made in an atmosphere of inert gas; the temperature, measured by a thermocouple in direct contact with the liquid metal, was kept constant (±0.1 °C). 

XRD patterns were collected at increasing temperatures with Mo-Kα radiation (**λ** = 0.07093 nm) in the 2Θ angular range 5–55° with steps of 0.05° and counting time of 5 s per step. Before each test run, liquid LBE alloy was thermally stabilized for 30 min. From each pattern, the radial distribution function (RDF) was determined with a procedure described in ref [12]. 

**Figure 1:**
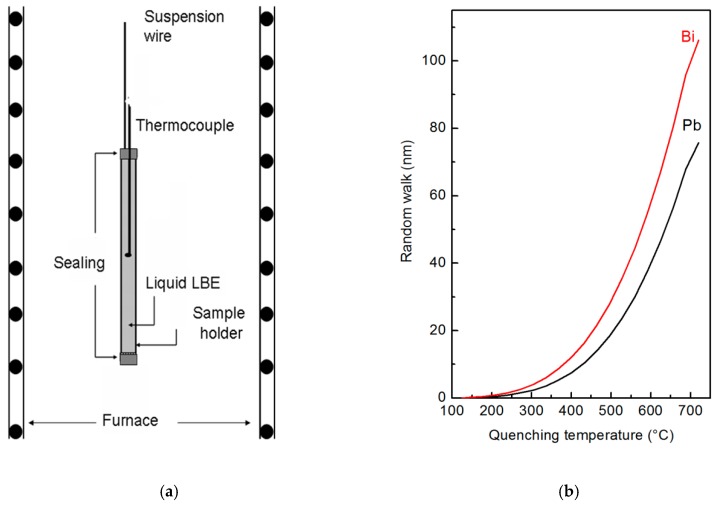
Sketch of the experimental set-up used for quenching liquid lead-bismuth eutectic (LBE) alloy (**a**) and random walk of Bi and Pb atoms in liquid LBE vs. the quenching temperature (**b**).

The samples used for XPS (manufacturer, city, country) and SPEM (manufacturer, city, country) measurements were prepared through rapid quenching of the liquid from different temperatures. The metal was preliminarily cast inside a thin-walled container of AISI 316L steel closed at an extremity, and a thermocouple was embedded into the metal. After solidification, the other extremity of the container has been sealed. A sketch of the sample holder and experimental set-up are displayed in Figure 1a. 

To study the characteristics of the liquid alloy in an extended temperature range, the experiments were carried out from 125 °C (eutectic temperature) to 720 °C. In each experiment, the sample holder was suspended in a vertical tubular furnace (manufacturer, city, country), heated at the selected temperature, thermally stabilized for 30 min and finally quenched in water. The lower end of furnace is open, so quenching was realized by cutting the suspension wire allowing the sample holder to fall in a water tank allocated under the furnace. Finally, the solid metal was extracted from the container, and its surface was examined through XPS and SPEM techniques. 

Cooling curves of the liquid alloy were measured in different positions inside the sample holder. Of course, the cooling rate depends on the distance from the wall of the sample holder, where the material experiences the fastest temperature change: It was ~520 °C s^−1^ at the centre of the sample and ~3200 °C s^−1^ at the surface. In the conditions of present experiments, the free random walk *W* of Bi and Pb atoms at the surface of liquid LBE was calculated vs. the quenching temperature (Figure 1b). *W* for the temperature *T* and time *t* is given by:

(1) $$W={(6Dt)}^{1/2},$$

where *D* is the diffusion coefficient. The diffusion coefficients of Pb and Bi atoms in liquid LBE were determined according to Yun Gao et al. [43]:

(2) $$D_{Pb/LBE}=4.0\times 10^{-4}\exp\left(\frac{-1.4\times 10^{4}}{RT}\right),$$

(3) $$D_{Bi/LBE}=4.0\times 10^{-4}\exp\left(\frac{-1.30\times 10^{4}}{RT}\right),$$

where *R* is the gas constant. Each value of the random walk plotted in Figure 1b has been calculated by integrating Equation (1) along the cooling curve in the range from the specific quenching temperature to 125 °C where solidification takes place. Diffusion in the solid-state is negligible with respect to that in the liquid, therefore, it was not considered.

The results in Figure 1b show that *W* is equal to 105 nm for Bi and 80 nm for Pb at the highest temperature examined here (720 °C); its value rapidly decreases with temperature. Therefore, XPS and SPEM measurements carried out on the free surface of the quenched samples describe a micro-chemical distribution close to the original one of the liquid alloys at high temperature. SPEM is used in present work to describe elemental segregation on a micro-scale, with the experimental limits reported in Figure 1b, while the heterogeneity on an atomic scale has been investigated by high temperature XRD measurements.

The photoemission measurements were carried out in a spectrometer Escalab 250Xi (Thermo Fisher Scientific Ltd, East Grinstead, UK) equipped with the monochromatic X-ray source and 6-channeltron detector. Before the measurements, all the samples were cleaned with pure ethanol. The surface of the samples was additionally cleaned in ultra-high vacuum by Ar^+^ ion sputtering at 2.0 keV energy. Experimental data were processed by the Avantage v.5 software (Thermo Fisher Scientific Ltd, East Grinstead, UK).

XPS measurements with high lateral resolution were performed by using the SPEM apparatus at the ESCA microscopy beamline of Elettra synchrotron in Trieste, Italy. Soft X-rays of synchrotron beam are focused to a diameter of around 150 nm by Fresnel zone plate optics, and the scanning is performed by raster movement of the sample under X-rays nanoprobe. Photoemission signal is collected by a PHOIBOS 100 hemispherical analyzer (SPECS GmbH, Berlin Germany) and registered by a 48-channel detector. This system can be used in both imaging (chemical maps) and spectroscopy (selected photoemission regions) modes, which were carried out at 667 eV photon energy with an energy resolution of 0.2 eV. Acquired chemical maps were processed by Igor v.6.3.2.3 and Matlab v.7.14 software (MathWorks, Cambridge, UK). More details on this technique have been reported elsewhere [44].

## 3. Results and Discussion

### 3.1. Structural Characterization 

The XRD patterns recorded at different temperatures were analyzed to determine the RDF curves. RDFs were calculated by using the following relationship:

(4) $$RDF=2\pi r^{2}\rho_{e}\sum_{UC}Z_{j}+\int_{0}^{Q\max}Qi(Q)e^{-\alpha^{2}Q^{2}}\sin rQdQ,$$

where *ρ_e_* is the mean electron density of the alloy, *Q* = 4*π* sin*θ*/*λ*, *i*(*Q*) the normalized diffracted intensity, *Z_j_* the atomic number of the atomic specie *j* (*j* = 1 *Pb*, *j* = 2 *Bi*), UC the composition unit. The *G*(*r*) curve is obtained by subtracting the parabolic contribution $2\pi r^{2}\rho_{e}\sum_{UC}Z_{j}$ from RDF. 

**Figure 2:**
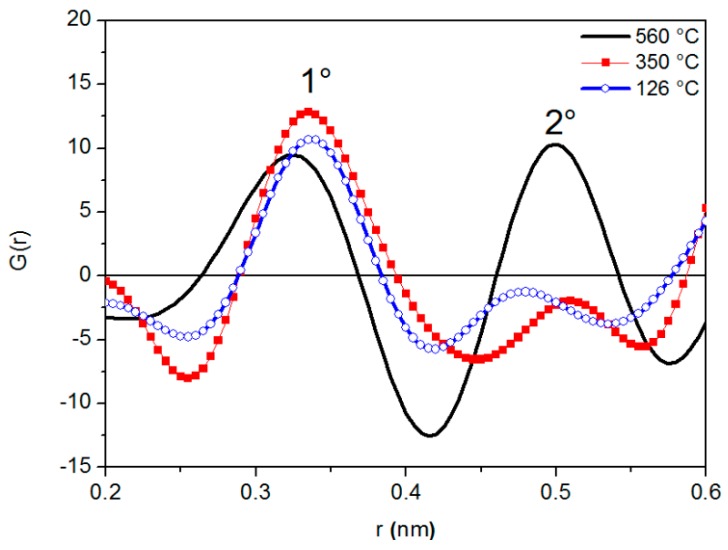
*G*(*r*) curves of liquid LBE at different temperatures.

Figure 2 shows *G*(*r*) curves at different temperatures. All of them exhibit two peaks with positions *r*_1_ and *r*_2_ (average radii of the first and second atomic shells surrounding a given atom): The arrangement of atoms in the liquid metal can be identified from the ratio *r*_2_/*r*_1_. As clearly shown in Figure 2, the variations of peak position are accompanied by changes in peak shape. 

The half width of the first RDF peak at 126 °C is a little wider than that recorded at a close temperature (140 °C) by Gudowski et al. [45] through neutron diffraction. Such discrepancy can be explained by considering the inherent characteristics of neutron and X-rays diffraction [46]: Neutrons exhibit a greater resolution in polyatomic systems because they are scattered by the nuclei; namely, there is essentially point scattering, and the scattering amplitudes do not vary with angle. 

**Figure 3:**
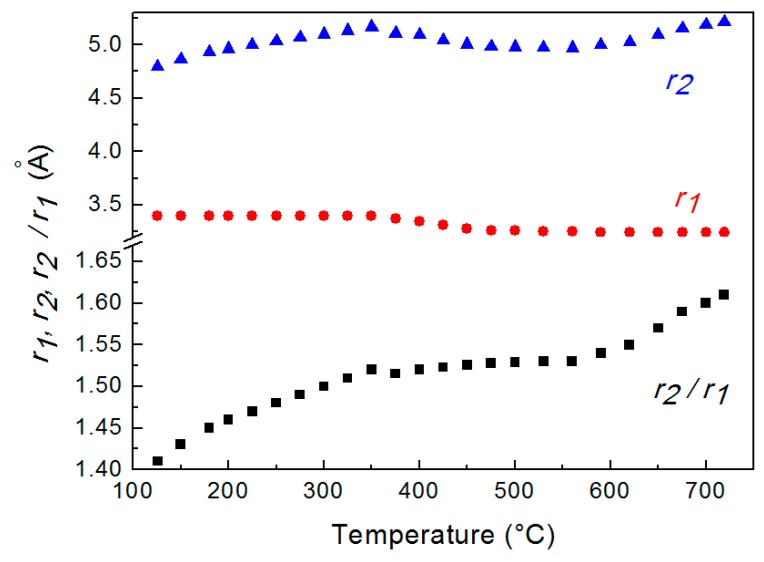
Average distances *r*_1_ and *r*_2_ of 1st and 2nd nearest neighbors determined from *G*(*r*) at different temperature and their ratio *r*_2_/*r*_1_. The scale is enlarged in the lower part of the graph to evidence *r*_2_/*r*_1_ variations.

Figure 3 displays the distances *r*_1_ and *r*_2_ determined from *G*(*r*) curves at different temperatures. The value of *r*_1_ is constant up to 350 °C, decreases from 350 °C to 520 °C and, finally is constant again. The ratio *r*_2_/*r*_1_, which describes the short-range order in the liquid metal, is also shown in Figure 3. 

**Figure 4:**
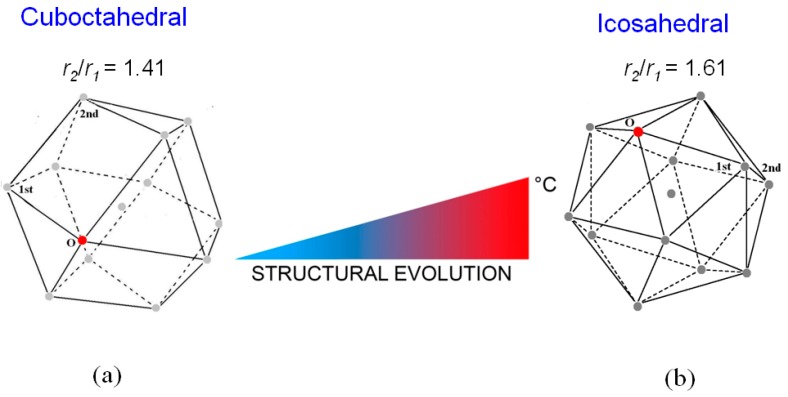
The structure of the liquid LBE alloy passes from the cuboctahedral configuration (**a**) to the icosahedral one (**b**) as temperature increases from 125 °C to 720 °C. The positions of 1st and 2nd nearest neighbors of a given atom in the position O are shown.

The trend of the ratio *r*_2_/*r*_1_ vs. temperature shows that just after melting *r*_2_/*r*_1_ is ~1.41 and increases till the value of ~1.61 at 720 °C; in the range from ~350 °C to 560 °C the ratio exhibits a plateau. This result indicates that the short-range order of liquid LBE gradually changes from a cuboctahedral configuration (the ratio is $\sqrt{2}$ ≅ 1.41) just after melting to an icosahedral one (the ratio corresponds to the golden ratio φ = 1.61) at 720 °C. Figure 4 displays the two structures of the liquid; in both configurations, the positions of 1st and 2nd nearest neighbors of a given atom in the position O are shown.

From the *r*_1_ values in Figure 4, the volumes of cuboctahedron at 125 °C and icosahedron at 720 °C result to be 92 Å^3^ and 78 Å^3^, respectively. In both configurations each atom is surrounded by 12 next neighbors; thus, the lower volume of the icosahedral structure involves a negative expansion of the interatomic distances as temperature increases. This behavior is anomalous since materials usually dilate when heated; however, a similar phenomenon was recently observed also by Lou et al. [47] in some pure metals (Al, Zn, Sn, In, Cu, Ag, Au and Ni).

The data of *r*_1_ and *r*_2_ determined from *G*(*r*) curves and plotted in Figure 3 are average values which do not account for the two atomic species, Bi and Pb. In fact, both 1st and 2nd peaks are the sum of three contributions coming from Pb–Pb, Bi–Bi and Pb–Bi pairs and *G*(*r*) peaks are the overlaps of these contributions which have been calculated through the following procedure. 

Around a given atom there are shells of neighbour atoms designed by *i*; *N_ij_* is the mean number of atoms in the shell *i* at a distance *r_ij_* from an atom of type *j.* The single contributions can be described by the pair functions *P_ij_*:

(5) $$P_{ij}(r)=\int_{0}^{Q\max_{i}}\frac{f_{j}f_{i}}{g^{2}(Q)}e^{-\alpha^{2}Q^{2}}\sin Qr_{ij}\sin QrdQ,$$

where *f_j_* and *f_i_* are the atomic scattering factors of the atoms forming the pair, *α*^2^*Q*^2^ is a factor of convergence introduced to avoid spurious oscillations along with the tails of the pair functions, while 1/*g*^2^(*Q*) is a sharpening factor. The function *g*(*Q*) decreases with increasing *Q* and has the value *g*(*Q*) = 1 at *Q* = 0. The function

(6) $$g(Q)=\frac{\sum_{UC}f_{j}}{\sum_{UC}Z_{j}},$$

has been used by us, but other suitable functions can be used as well. 

The RDF is expressed as:

(7) $$RDF=\sum_{UC}\sum_{i}\frac{N_{ij}}{r_{ij}}P_{ij}(r).$$

Equation (8), obtained by combining Equations (4) and (7), enables to analyze the RDF curves:

(8) $$\sum_{UC}\sum_{i}\frac{N_{ij}}{r_{ij}}P_{ij}(r)=2\pi^{2}r\rho_{e}\sum_{UC}Z_{j}+\int_{0}^{Q\max}Qi(Q)e^{-\alpha^{2}Q^{2}}\sin rQdQ.$$

**Figure 5:**
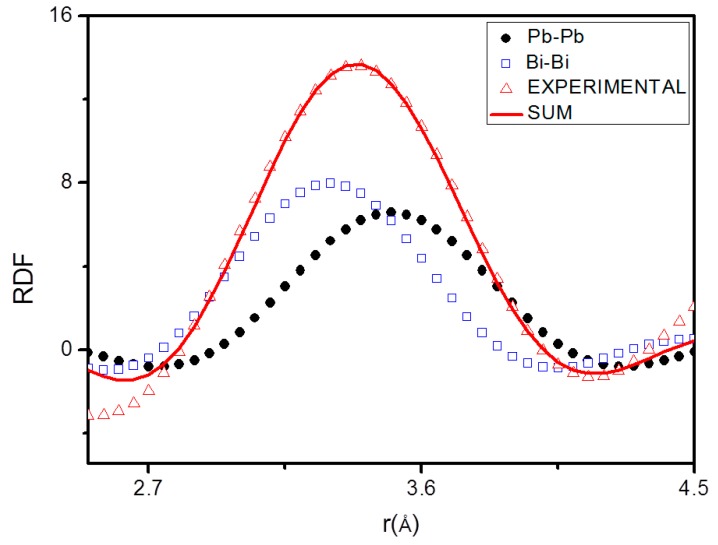
Fitting of the first radial distribution function (RDF) peak at 126 °C peak by means of the pair functions.

The pair functions *P_ij_*(*r*) have been determined for each of the *r_ij_*, then used to find the *N_ij_* values which bring the left-hand side of Equation (8) into the best fit with the right-hand side. An example of fitting of the first RDF peak by pair functions *P_ij_*(*r*) is shown in Figure 5. 

**Figure 6:**
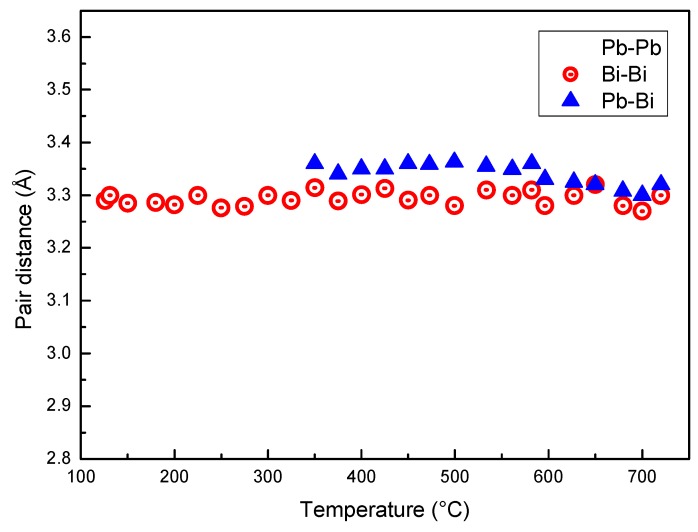
Distance between Pb–Pb, Bi–Bi and Pb–Bi pairs vs. temperature.

Figure 6 displays the distance between Pb–Pb, Bi–Bi and Pb–Bi pairs vs. temperature. At the eutectic temperature, solid LBE consists of two phases, Bi and the hexagonal β phase (cell parameters a = 0.35058 nm, c = 0.57959 nm) that has a Bi content of 41.8 at %. The distance between the next neighbors in the β phase is a ~0.35 nm and substantially corresponds to that of Pb–Pb pairs just after melting. In fact, Pb is present only in the β phase, and the distance of Pb–Pb pairs in the melt is close to that of atoms in the solid. The Pb–Pb distance slightly decreases up to 350 °C; then, a remarkable drop is observed in the range 350–520 °C, while above 520 °C its value is nearly constant (~0.29 nm). On the contrary, Pb–Bi and Bi–Bi distances substantially do not change with temperature. Therefore, the variations of *r*_1_ displayed in Figure 3 depends on the bonds between Pb atoms in the melt and on the change of the distance of Pb–Pb pairs.

**Figure 7:**
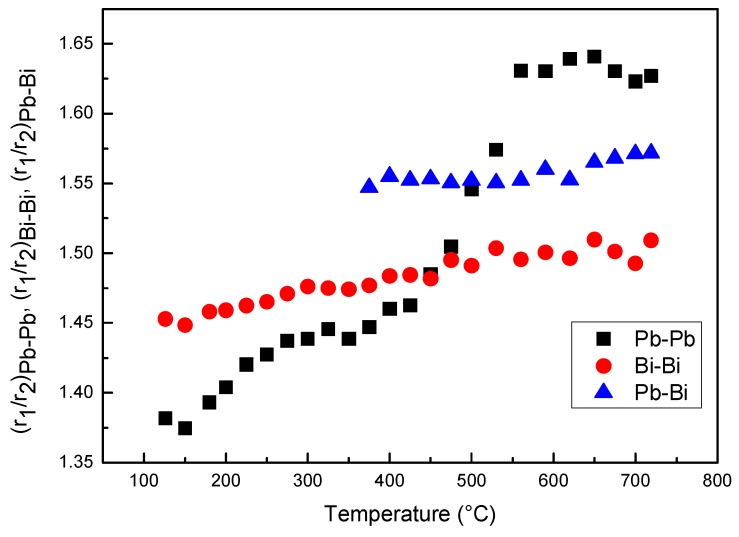
Ratios *r*_2_/*r*_1_ of Bi–Bi, Pb–Pb and Pb–Bi pairs vs. temperature.

From the fitting of the 1st and 2nd peaks of RDF curves the ratios *r*_2_/*r*_1_ of Pb–Pb, Bi–Bi and Pb–Bi pairs vs. temperature have been determined (Figure 7). The ratios of Pb–Bi and Bi–Bi pairs slightly increase with temperature, whereas, that of Pb–Pb pairs exhibits a more pronounced variation from ~1.39 to ~1.62, two values very close to the ideal cubooctahedral and icosahedral configurations, respectively. 

**Figure 8:**
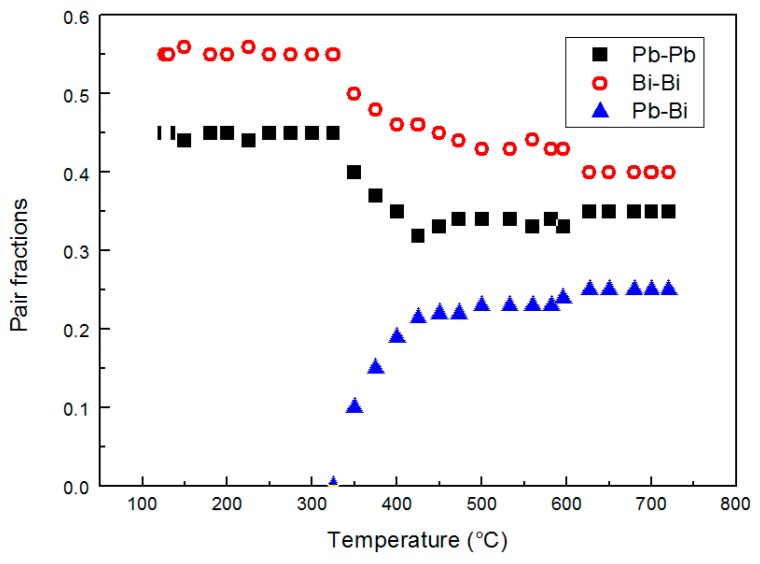
The relative amount of Bi–Bi, Pb–Bi and Pb–Pb pairs vs. temperature.

The relative amounts of Bi–Bi, Pb–Pb and Pb–Bi pairs vs. temperature are plotted in Figure 8. 

The Pb–Bi pairs are absent from the melting point to 350 °C; then, their contribution becomes increasingly important with temperature (Figure 8).

Since LBE consists of 44.1 at % of Pb and 55.9 at % of Bi the following relationships can be written for the fractions *X* of Pb–Pb, Bi–Bi and Pb–Bi pairs:

(9) $$X_{Pb-Pb}+\frac{1}{2}X_{Pb-Bi}=0.441,$$

(10) $$X_{Bi-Bi}+\frac{1}{2}X_{Pb-Bi}=0.559.$$

Therefore, the increasing number of$X_{Pb-Pb}+\frac{1}{2}X_{Pb-Bi}=0.441$ Pb–Bi pairs above 350 °C involves a decrease of Bi–Bi and Pb–Pb pairs and consequently corresponds to a more homogeneous distribution of the elements in the alloy. It is noteworthy to observe that such distribution of Bi and Pb atoms is not fully random (around 0.19 for Pb–Pb, 0.31 for Bi–Bi, and 0.49 for Pb–Bi/Bi-Pb); from the asymptotic trends in Figure 8 the distribution is substantially stable above ~450 °C, at least in the temperature range examined here, and is affected by the tendency of Pb and Bi atoms to bond with atoms of the same type that hinders to reach a completely random distribution. 

**Figure 9:**
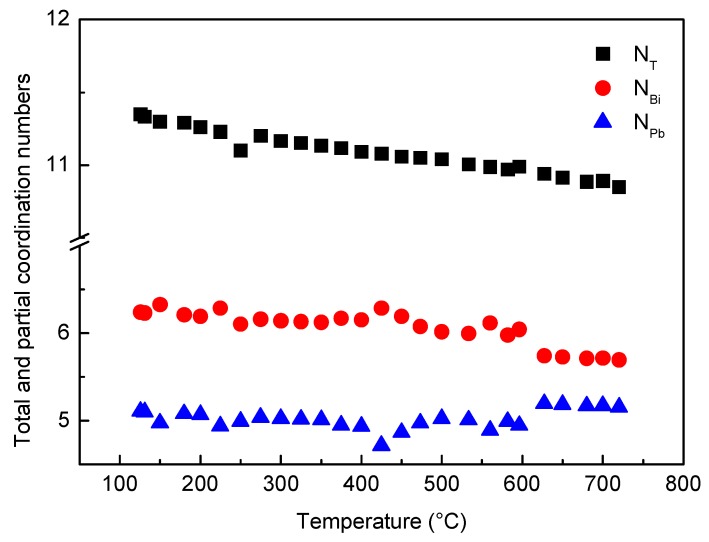
Total (N_T_) and partial coordination numbers of Bi (N_Bi_) and Pb (N_Pb_) vs. temperature.

The total coordination number N_T_ calculated from RDF curves vs. temperature is plotted in Figure 9; the value is about 11, and its trend continuously decreases without abrupt variations as temperature increases. Such result is in agreement with the monotonical density decrease of liquid LBE obtained from experimental measurements [48] and ab initio molecular dynamics simulations of Song et al. [49] and Han et al. [50].

In Figure 9, the partial coordination numbers of Bi (N_Bi_) and Pb (N_Pb_) are also reported.

Owing to its interest for nuclear applications, the characteristics of liquid LBE have been studied by many investigators and a lot of data are available in the literature. Its behavior is quite puzzling because some properties exhibit a continuous trend vs. temperature, such as density, while other properties show a discontinuity. Mechanical Spectroscopy tests made by Zu et al. [33] and some of the present authors [38] showed a damping maximum accompanied by a huge change of dynamic modulus. These results, clearly connected to a change of the forces between atoms, are in agreement with the variations of the relative number of Pb–Pb, Pb–Bi and Bi–Bi pairs described in this work. On the other hand, variations of the pair distribution modify the electron structure of liquid LBE and may lead to the anomaly of the electrical conductivity σ(T) curve observed by some investigators [39,40]. However, the redistribution of chemical elements and the structural change do not involve an abrupt change of density because the trend of coordination number vs. temperature is continuously decreasing. 

In a future work, advanced computational techniques (e.g., see Reference [51,52,53]) and topological analysis, including Voronoi analysis or CNA (common neighbor analysis), will be adopted to analyze the structural transformations in the liquid LBE. Such an approach will permit to get further information to compare with present results. 

### 3.2. Chemical Characterization

The surface chemical composition of LBE alloy quenched from different temperatures was investigated by XPS. This analysis technique is surface sensitive; therefore, it is typically employed to investigate the chemical composition of the first layers (~4–5 nm) of materials in a solid-state. The distribution of alloying elements on the surface, which experiences the highest cooling rate, is not far from that present in the original liquid; therefore, the results give information useful to understand the chemical homogeneity of liquid LBE. In first approximation, it can be assumed that the liquid alloy is frozen in its original state.

In all the samples, XPS results reveal the presence of Bi, C, Pb and O. Carbon is due to ambient contamination and is removed after few cycles of ion sputtering (Ar^+^), whereas, the presence of O is related to the formation of Bi and Pb oxides. The spectra of Bi 4f and Pb 4f were processed by a peak-fitting routine. This analysis revealed that Pb 4f signal is composed of two Pb 4f_7/2_ peaks with binding energy BE = 136.7 and 138.4 eV, which have been identified as metal and PbO_2_, respectively. Similarly, the Bi 4f_7/2_ signal is composed of two peaks, localized at BE = 156.0 and 158.5 eV, which correspond to metal and Bi_2_O_3_, respectively. Since the presence of oxides can be misleading in the study of segregation phenomena in liquid LBE alloy, Ar^+^ ion sputtering was employed to remove the topmost oxidized layers. 

In all the examined samples, the XPS measurements made on the surface and on cross-section did not register the signals of Cr, Ni and Fe (i.e., the main elements of AISI 316L steel), therefore, the contamination of the melt by corrosion products coming from the container can be considered negligible.

On the basis of XPS results, also the SPEM measurements were performed after the removal of 20 nm overlayer from the surface by ion sputtering.

**Figure 10:**
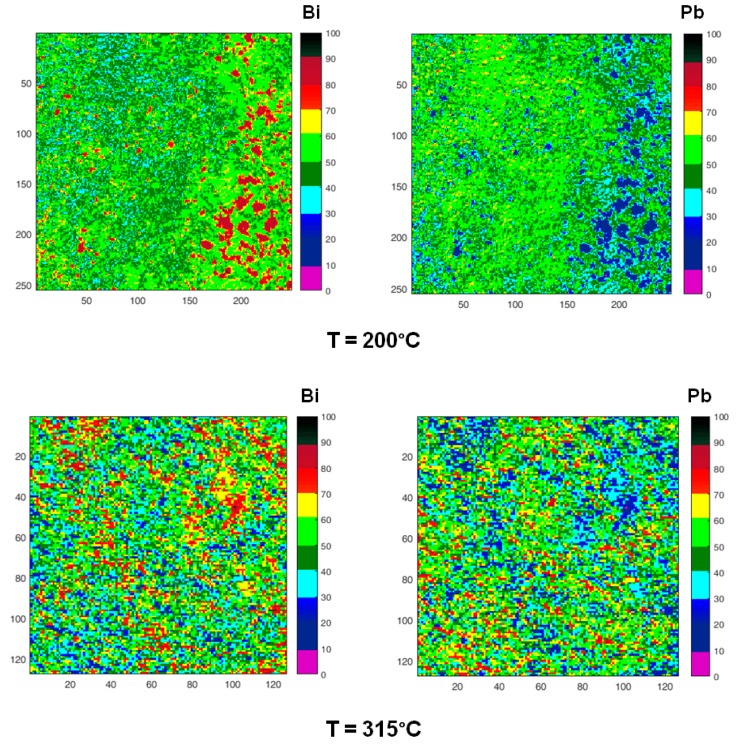

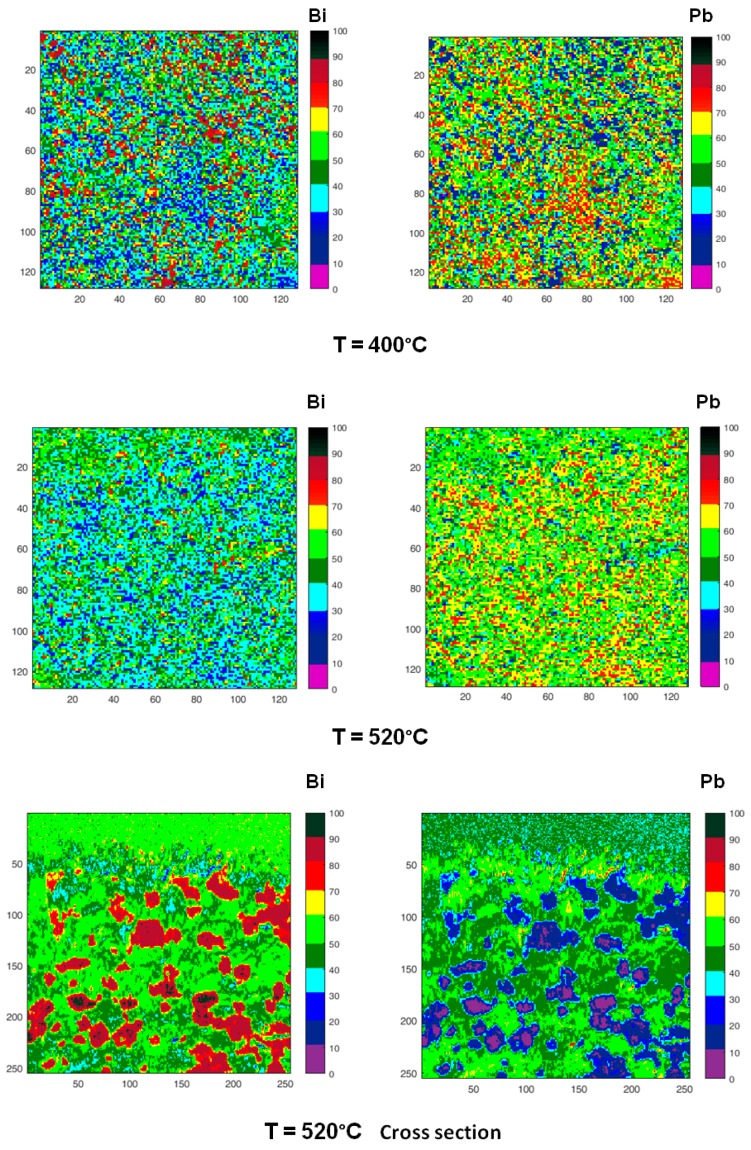
Chemical maps of Bi (left column) and Pb (right column) collected on the sample surface after quenching from increasing temperatures (200, 315, 400, 520 °C). The last two micrographs have been collected on the cross-section of the sample quenched from 520 °C. All images show an area 100 µm × 100 µm except those recorded on the cross-section (50 µm × 50 µm). Numbers on the scale axis indicate the acquisition channels.

SPEM is a very surface sensitive microscopy, which permits to observe the chemical distribution of the elements at high lateral resolution (~100 nm). To study the micro-chemical distribution, SPEM measurements were performed by collecting maps of the surface area (100 µm ×100 µm) on quenched samples. These maps (Figure 10) are composed of 128 × 128 or 256 × 256 pixels, where each pixel is the intensity of the selected photoemission signal (Pb 4f_7/2_ and Bi 4f_7/2_), mediated on 48-channels [54]. Since the photoemission signal depends on the sample height, i.e., the distance between the sample and analyser input lens, the chemical maps of SPEM can be influenced by sample roughness [54]. These topography effects have been eliminated from the chemical maps by using (peak–background)/background routine of Igor software. The local concentration of the elements in the maps is described by the colour scale on the right in Figure 10. 

Zones enriched in Bi are detected in the samples quenched from temperatures in the range of 125–315 °C, their size is of the order of few microns at 125 °C and tends to decrease with temperature. At 400 °C, some residual zones rich in Bi are still visible with size ≤ 5 μm, while at 520 °C Pb and Bi are homogeneously distributed.

The chemical map acquired on the cross-section of the sample quenched from 520 °C is quite different from that recorded on the surface: it displays large clusters rich in Bi on the inner part of the sample, where the material experiences the lower cooling rate. 

**Figure 11:**
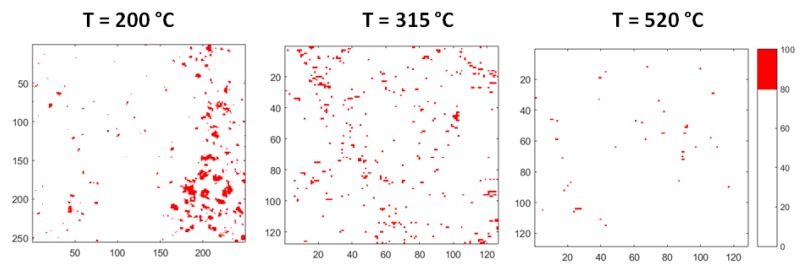
Evolution of the areas rich in Bi: The images are obtained by plotting in red colour only the zones where Bi content is in the range of 80–100 at %.

Figure 11 displays the evolution of the areas rich in Bi. The images are obtained from corresponding maps of Figure 10 by plotting in red colour only the zones, where Bi content is in the range of 80–100 at %. It is observed that the aggregates rich in Bi dissolve as temperature increases: At 315 °C only a few small areas remain, and the process is substantially completed at 520 °C. 

**Figure 12:**
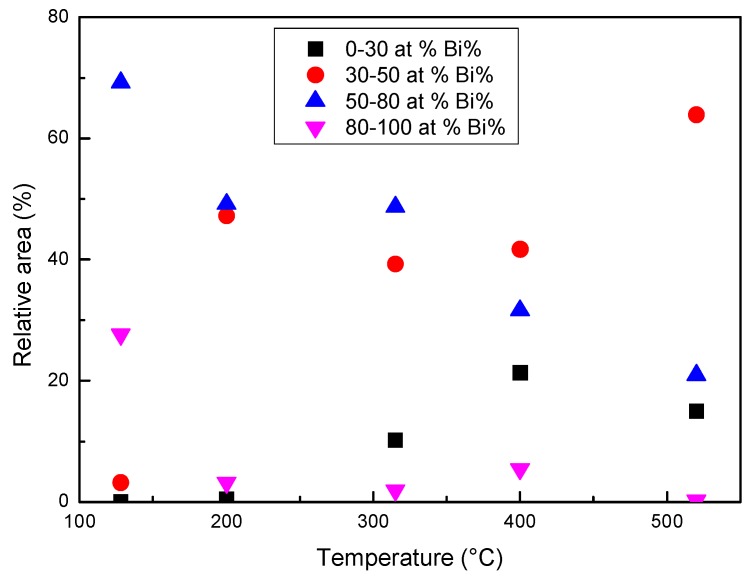
The relative area of the pixels with Bi in four different ranges (at %) determined from the images in Figure 10.

When the temperature reaches or exceeds 315 °C, a diffused interface is observed between the zones with different composition or at least it is not possible to identify specific interfaces with the lateral resolution of the images in Figure 10, where each pixel represents the mean elemental content of 0.8 × 0.8 or 0.4 × 0.4 μm^2^. In order to describe the process of homogenization, the relative areas of the pixels with Bi content in four different ranges (at %) have been plotted vs. temperature in Figure 12. The ranges of 80–100 and 30–50 at % represent the Bi-rich zones, which progressively dissolve, and the areas with a composition tending to the average one, which is about 70% at 520 °C.

SPEM maps confirm the results of HT-XRD and provide additional information on the processes occurring in liquid LBE on an atomic and micro-scale. From melting point to 350 °C, the atoms of the same species tend to be bound together (Pb–Bi pairs are not present) and at the lowest temperature they form clusters, which progressively dissolve as temperature increases and only a few residuals remain at 315 °C (Figure 11 and Figure 12). When the temperature exceeds 350 °C, a process of homogenization also starts on an atomic scale: The fraction of Pb–Bi pairs increases at the expense of the Bi–Bi and Pb–Pb ones and the clusters break up and completely disappear at 520 °C. The behavior of liquid LBE suggests that homogenization on a micro-scale is the condition for a general re-arrangement on an atomic scale leading to the formation of Pb–Bi pairs. 

**Figure 13:**
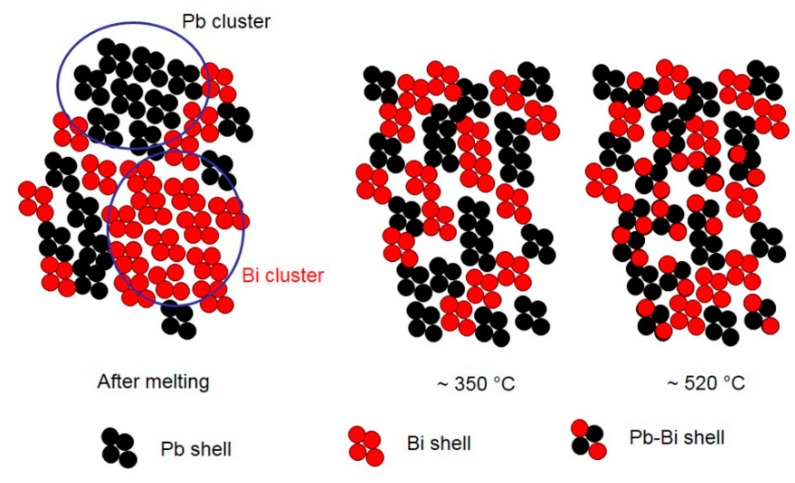
Schematic view of the atomic and micro-scale transformations occurring in liquid LBE alloy at increasing temperature.

A schematic view of the atomic and micro-scale transformations occurring in liquid LBE alloy at increasing temperature is displayed in Figure 13. The figure is just a sketch for illustrating the chemical evolution of the system and does not quantitatively account for the local atom environment.

In conclusion, chemical heterogeneity in liquid LBE occurs on both micro- and atomic scale. The heterogeneity on an atomic scale has been studied by means of high temperature XRD, and the results are summarized in Figure 8 showing the distribution of Pb–Bi, Pb–Pb, and Bi–Bi pairs vs. temperature. Owing to insufficient spatial resolution SPEM and XPS are not able to evidence such type of heterogeneity; however, segregation takes place also on a micro-scale and SPEM has been used to realize chemical maps which describe the phenomenon. The comparison of the results obtained by XRD and SPEM leads to conclude that the homogenization at the micro-scale seems to be the condition for the occurrence of that on an atomic scale.

## 4. Conclusions

The results of the present investigation carried out on liquid LBE from the eutectic temperature to 720 °C can be summarized as follows.

1. After melting, the short-range order in liquid metal corresponds to a cuboctahedral arrangement of atoms that progressively evolves towards an icosahedral one as temperature increases. This structural transformation involves a negative expansion of the interatomic distances.
2. This process is accompanied by variations of chemical distribution at the micro- and atomic scale, which take place in the temperature range of 350–520 °C.
3. A change of the relative number of Pb–Pb, Pb–Bi and Bi–Bi pairs is observed on an atomic scale. The Pb–Bi pairs are detected only at temperatures above ~350 °C and their fraction progressively increases, resulting in a more homogeneous distribution of the elements in the alloy.
4. The negative expansion of the interatomic distances in liquid LBE is mainly related to the atomic distance in Pb–Pb pairs, which drops from 350 to 520 °C, while Pb–Bi and Bi–Bi distances substantially do not change with temperature.
5. The SPEM elemental maps, collected on the alloy surface after quenching from different temperatures, confirm a process of chemical homogenization on a micro-scale. The clusters rich in Bi and Pb are observed after alloy melting. They progressively dissolve as the temperature increases: Only a few residuals remain at 315 °C, and no more clusters are detected a 520 °C.
6. Homogenization at the micro-scale seems to be the condition for the occurrence of that on an atomic scale.
